# Clinical stem cell therapy in oral and craniofacial bone regeneration: a systematic review and meta-analysis

**DOI:** 10.3389/fbioe.2026.1677400

**Published:** 2026-01-23

**Authors:** Parham Hazrati, Abdulmohsen Alanazi, Abdusalam E. Alrmali, Pablo Galindo-Fernandez, Hazar Kassem, Darnell Kaigler

**Affiliations:** 1 Department of Periodontics and Oral Medicine, University of Michigan School of Dentistry, Ann Arbor, MI, United States; 2 Department of Oral Medicine, Oral Pathology, Oral and Maxillofacial Surgery, University of Tripoli School of Dentistry, Tripoli, Libya; 3 Department of Biomedical Engineering, College of Engineering, University of Michigan, Ann Arbor, MI, United States

**Keywords:** stem cells, bone regeneration, dental implants, maxillofacial injuries, maxillofacial abnormalities, alveolar bone grafting, sinus floor augmentation

## Abstract

Craniomaxillofacial bone regeneration poses significant clinical challenges due to the anatomical complexity of this region and the inherent limitations of conventional reconstructive techniques. Stem cell-based therapies have emerged as a promising alternative in that stem cells harness the capacities of multilineage differentiation and paracrine signaling to enhance tissue regeneration. Nonetheless, the overall clinical efficacy of stem cell therapy remains a subject of debate. In this systematic review and meta-analysis, we aimed to comprehensively evaluate the safety and effectiveness of stem cell therapy in oral and craniofacial bone regeneration. A comprehensive search of PubMed/MEDLINE, Scopus, Embase, and Web of Science was conducted in July 2024, identifying 59 eligible prospective studies—including randomized controlled trials (RCTs), controlled clinical trials and single-arm studies—involving more than five participants each. Risk of bias was assessed using the Cochrane RoB 2 tool for randomized studies and ROBINS-I for non-randomized studies. The included studies encompassed a broad range of surgical indications, such as alveolar cleft repair, alveolar ridge augmentation, sinus floor augmentation, periodontal defect regeneration, mandibular fracture management, pathological bone defect repair, and temporomandibular joint disorders. Over three-quarters of studies utilized bone marrow aspirate (BMA) and/or mesenchymal stem cells (MSCs), either alone or combined with biomaterial scaffolds. Across diverse procedures, stem cell therapy was associated with clinical and histological benefits, especially in the quality and maturity of regenerated bone. Meta-analysis showed that the addition of stem cells significantly improved the histologic quality of regenerated bone (*p* = 0.0446), although this enhancement was not evident in radiographic assessments (*p* = 0.1094). Additionally, meta-analyses demonstrated that stem cell therapy did not result in significant improvements in periodontal clinical attachment level (CAL) gain (*p* = 0.0730) or linear bone height (*p* = 0.1858) and width (*p* = 0.8323) compared to conventional treatments. Notably, volumetric (3D) radiographic assessments indicated significantly enhanced bone volume regeneration in stem cell-treated groups (*p* = 0.0218). Overall, stem cell therapy shows promising potential in craniomaxillofacial bone regeneration, but heterogeneity among studies underscores the need for further standardized clinical trials to establish definitive benefits, as well as consistent reporting.

**Systematic review registration:**

The protocol of this systematic review was registered on PROSPERO with the ID CRD42024619352.

## 1 Introduction

Achieving predictable craniomaxillofacial bone regeneration remains a significant challenge in reconstructive surgery and regenerative dentistry. Defects arising from trauma, congenital disorders, infections, or degenerative conditions present significant clinical complexities, largely due to the region’s highly variable anatomical features (Kinoshita and Maeda, 2013). On a global scale, head injuries affect approximately 47 million individuals, with 21.6 million new cases reported each year (James et al., 2018). Bone loss resulting from periodontal disease, oral cancer, tooth extraction, infection, or trauma can undermine facial appearance and essential oral functions such as chewing, speaking, and nutrition, thereby greatly diminishing patients’ quality of life (Bodic et al., 2005).

Conventional reconstructive treatment approaches face significant limitations. Autologous bone grafts, considered the gold standard for reconstruction, exhibit 20%–30% donor-site morbidity (including chronic pain and infection) and suffer up to 60% resorption in non-vascularized grafts (Thalakiriyawa and Dissanayaka, 2024). Guided tissue and bone regeneration (GTR/GBR) is compromised by material deficiencies: non-resorbable membranes [e.g., expanded polytetrafluoroethylene (ePTFE)] necessitate secondary removal surgeries (Thalakiriyawa and Dissanayaka, 2024), collagen membranes degrade too rapidly (<4 weeks) to support complete healing (Elsalanty and Genecov, 2009), and synthetic alternatives frequently provoke foreign-body reactions (Gil et al., 2023). Biomaterials such as hydroxyapatite and bioactive glass lack essential biological signals for functional interface regeneration (e.g., Sharpey’s fiber insertion), while growth factors like BMP-2 paradoxically suppress cementogenesis and induce root resorption (Aghali, 2021).

The craniomaxillofacial region encompasses diverse tissues — bone, mucosa, periodontal ligament (PDL), and cementum — each with distinct structural and biological properties. Specifically, periodontal regeneration proves exceptionally complex due to the periodontium’s intricate architecture, particularly its dual-tissue interfaces like the alveolar bone–PDL and PDL–cementum junctions (Majzoub et al., 2020). True regeneration requires not only tissue restoration but also seamless integration of these interfaces to reestablish functional integrity. Compounding these challenges, the oral environment introduces microbial contamination, masticatory forces, and restricted vascularization in defect sites (Cho et al., 2021).

Stem cell-based therapies represent a transformative approach in craniomaxillofacial and periodontal regeneration, offering solutions to limitations of traditional methods like donor-site morbidity, unpredictable resorption, and inadequate bioactivity (Huang et al., 2024). Unlike conventional grafts or biomaterials, stem cells exhibit the properties of self-renewal and multilineage differentiation, enabling regeneration of complex tissue architectures (Nguyen-Thi et al., 2023; Bartold et al., 2006). Mesenchymal stem cells (MSCs), for instance, can differentiate into osteoblasts for bone formation, cementoblasts for root cementum deposition, and fibroblasts for PDL fiber synthesis (Huang et al., 2009; Nguyen-Thi et al., 2023). This inherent plasticity allows simultaneous regeneration of critical interfaces (e.g., bone–PDL–cementum complex), which traditional materials often fail to restore (Qu et al., 2019).

Critical research gaps persist in regenerative dentistry, including technical limitations, procedural invasiveness, and prognostic uncertainty. Craniofacial bone regeneration is highly dependent on use of materials to treat defects in calvaria, facial bones, and temporomandibular joints (TMJ); despite some degree of functional restoration using these materials, anatomic and functional restoration often remains unrealized (Tevlin et al., 2014). Current bone biomaterials face challenges like premature resorption/persistence and limited efficacy in large/uncontained defects (Latimer et al., 2021; Dzobo et al., 2018). Since 2000, bioengineering advances have accelerated, spanning stem cell therapies (Kaigler et al., 2013b; Iwata et al., 2015; Gómez-Barrena et al., 2011; Queiroz et al., 2021).

The integration of stem cells with advanced biomaterial scaffolds further amplifies their regenerative potential. Biomimetic scaffolds can be engineered to closely mimic the composition and architecture of the native extracellular matrix, providing both structural support and critical cues for cell attachment, proliferation, and differentiation (Davidopoulou et al., 2024; Chen et al., 2024). Recent innovations, such as three-dimensional (3D) printing and electrospinning, have enabled the fabrication of patient-specific scaffolds with precisely controlled porosity, fiber alignment, and degradation profiles. These features not only facilitate the spatial organization of multiple cell types and the delivery of growth factors but also allow for the reconstruction of complex, multi-tissue interfaces that are essential for functional regeneration in the craniomaxillofacial region (Chen et al., 2024). For example, 3D-printed polycaprolactone scaffolds with microchannels have been shown to guide the alignment of periodontal ligament fibers, while smart hydrogels can provide sustained release of growth factors like fibroblast growth factor 2 (FGF-2) to promote angiogenesis and tissue integration over extended periods.

Despite these advances in biomaterial design and engineering, several challenges remain before stem cell-based therapies can be predictably integrated with the use of these biomaterials and ultimately translated into clinical practice. Ensuring the consistent quality and viability of stem cells during isolation, expansion, and delivery is critical for achieving reproducible outcomes. The development of delivery systems that can precisely target stem cells to defect sites, enhance their retention and survival, and promote their integration with host tissues remains an area of active research. Furthermore, the long-term stability and functional integration of regenerated tissues—particularly in the mechanically dynamic and microbiologically complex environment of the oral cavity—are essential for achieving durable clinical success. Patient-specific factors, such as age, systemic health conditions (including diabetes and immunosuppression), and lifestyle habits like smoking, can significantly influence the regenerative capacity of both transplanted and endogenous cells, adding an additional layer of complexity to treatment planning and outcome prediction (Shaikh et al., 2022; Huang et al., 2024).

This systematic review aims to evaluate the current evidence on the efficacy and safety of stem cell-based therapies for craniomaxillofacial and periodontal regeneration. By analyzing studies utilizing various stem cell sources and delivery methods across a range of clinical applications in oral and craniofacial regenerative procedures, this review seeks to provide a comprehensive overview of their potential benefits and limitations. Particular attention will be given to key clinical and histological outcomes such as new bone formation, clinical attachment level (CAL) gain in periodontal defects, vascularization of engineered bone, and long-term dimensional stability of regenerated tissues. Furthermore, this review will explore emerging trends in stem cell research—including advanced biomaterial design—that hold promise for advancing the field toward more predictable clinical outcomes.

## 2 Materials and methods

### 2.1 Protocol and registration

This systematic review and meta-analysis was conducted in accordance with the Preferred Reporting Items for Systematic Reviews and Meta-Analyses (PRISMA) 2020 guidelines, as detailed in Supplementary Tables S1, S2 (Matthew et al., 2021). The protocol for this review was prospectively registered in the International Prospective Register of Systematic Reviews (PROSPERO) under the reference number CRD42024619352.

### 2.2 PICOST framework and focus question

The following elements were used in formulating the focus question of current review:

Population: Patients (humans) with congenital or acquired skeletal defect or deficiency in the maxilla or mandible.
Intervention: Application of stem cells or autogenous biologics containing stem cells in surgical regenerative approaches.
Comparison: Other regenerative approaches not involving stem cell therapy or none.
Outcomes: Primarily, the quantity or quality of the regenerated bone.
Study design: Any human prospective clinical study with equal to or more than 5 patients, such as prospective randomized or non-randomized controlled clinical trials, and single arm pre-post studies with a proper protocol.
Timeframe: Articles published at any point in time.

Based on the mentioned framework, the following focused question was considered:

“In prospective clinical studies including at least five patients with congenital or acquired maxillofacial skeletal defects, what is the effect of applying stem cells or stem cell-containing autogenous biologics in surgical regenerative approaches, compared to other regenerative methods not involving stem cells, in terms of the quantity and/or quality of regenerated bone?”

### 2.3 Search strategy

A comprehensive literature search was conducted across PubMed/MEDLINE, Scopus, Web of Science, and Embase electronic databases. The specific search queries used for each database are detailed in Table 1. All articles identified through the search queries and published or indexed in these databases up to 27 July 2024, were retrieved without any restrictions on publication type, language, or year of publication. In addition to reviewing the reference lists of articles deemed eligible for inclusion, issues of the following journals published since 2000 were also manually examined: *British Journal of Oral and Maxillofacial Surgery, Clinical Advances in Periodontics, Clinical Implant Dentistry and Related Research, Clinical Oral Implants Research, Clinical Oral Investigations, International Journal of Oral and Maxillofacial Implants, International Journal of Oral and Maxillofacial Surgery, International Journal of Oral Implantology, International Journal of Periodontics and Restorative Dentistry, Journal of Clinical Periodontology, Journal of Craniofacial Surgery, Journal of Cranio-Maxillofacial Surgery, Journal of Dental Research, Journal of Maxillofacial and Oral Surgery, Journal of Oral and Maxillofacial Surgery, Journal of Oral Implantology, Journal of Oral Rehabilitation, Journal of Periodontology, Journal of Stomatology, Oral and Maxillofacial Surgery,* and *Oral and Maxillofacial Surgery.*

**TABLE 1:** Search queries.

Database	Date	Search query	Result
Embase	27 Jul 2024	((oral OR dental) AND implant* OR (sinus AND (lift OR elevat* OR augment*)) OR (bone AND (graft OR regenerat* OR reconstruct* OR augment* OR preserv*))) AND stem AND cell AND (maxillofac* OR craniofac* OR facial OR alveol* OR mandibul* OR maxilla* OR socket) NOT review NOT animal NOT vitro	1,375
Web of Science	27 Jul 2024	TS = (((oral OR dental) AND implant* OR (sinus AND (lift OR elevat* OR augment*)) OR (bone AND (graft OR regenerat* OR reconstruct* OR augment* OR preserv*))) AND stem AND cell AND (maxillofac* OR craniofac* OR facial OR alveol* OR mandibul* OR maxilla* OR socket) NOT review NOT animal NOT vitro)	1,286
Scopus	27 Jul 2024	TITLE-ABS-KEY ((((Oral OR Dental) AND Implant*) OR (Sinus AND (Lift OR Elevat* OR Augment*)) OR (Bone AND (Graft OR Regenerat* OR Reconstruct* OR Augment* OR Preserv*))) AND (Stem AND Cell) AND (Maxillofac* OR Craniofac* OR Facial OR Alveol* OR Mandibul* OR Maxilla”* OR Socket) AND NOT (Animal OR Review OR Vitro))	841
PubMed/MEDLINE	27 Jul 2024	((((((“Oral” [Title/Abstract] OR “Dental” [Title/Abstract]) AND “implant*” [Title/Abstract]) OR “Dental Implants” [MeSH Terms] OR (“Sinus floor augmentation” [MeSH Terms] OR (“Sinus” [Title/Abstract] AND (“Lift” [Title/Abstract] OR “elevat*” [Title/Abstract] OR “augment*” [Title/Abstract]))) OR (“Bone” [Title/Abstract] AND (“Graft” [Title/Abstract] OR “regenerat*” [Title/Abstract] OR “reconstruct*” [Title/Abstract] OR “augment*” [Title/Abstract] OR “preserv*” [Title/Abstract]))) AND (“Stem Cells” [MeSH Terms] OR “Stem Cell” [Title/Abstract]) AND (“maxillofac*” [Title/Abstract] OR “craniofac*” [Title/Abstract] OR “Facial” [Title/Abstract] OR “alveol*” [Title/Abstract] OR “mandibul*” [Title/Abstract] OR “maxilla*” [Title/Abstract] OR “socket” [Title/Abstract])) NOT “Animal” [Title/Abstract]) NOT “Review” [Title/Abstract]) NOT “vitro” [Title/Abstract]	708

### 2.4 Eligibility criteria

#### 2.4.1 Type of studies

Randomized controlled trials (RCTs), controlled (nonrandomized) clinical trials (CCTs), and single-arm prospective studies with equal to or more than 5 patients, were considered eligible. On the other hand, case reports, single-arm prospective studies with less than 5 patients, conference abstracts, protocols, hypothesis articles, and reviews were excluded.

#### 2.4.2 Type of participants

Participants with congenital or acquired craniomaxillofacial bone deformities or defects, including alveolar ridge defects, cleft palate, TMJ disorders, were considered eligible. Cases of soft tissue defects unaccompanied by bone defects or deformities were deemed ineligible, for example cleft lip cases without alveolar or palatal cleft.

#### 2.4.3 Type of intervention

Both cultured and expanded stem cells, as well as aspirates containing stem cells, were considered eligible for inclusion. In contrast, autologous platelet concentrates—such as platelet-rich fibrin (PRF), platelet-rich plasma (PRP), and concentrated growth factor (CGF)—were excluded.

#### 2.4.4 Type of outcome measure

Primary outcomes included quality and quantity of the regenerated bone. Secondary outcomes encompassed patient reported outcomes and periodontal CAL gain.

### 2.5 Study screening and data extraction

The initial screening involved evaluating the titles and abstracts of studies based on the specified eligibility criteria. Following this step, the full texts of selected articles were obtained for a more comprehensive review. Both title/abstract and full-text screening processes were independently conducted by two reviewers (A.A. and H.K.), and any differences in their assessments were resolved through discussion with a third expert reviewer (A.E.A.). This collaborative approach aimed to minimize bias and maintain a high standard of data integrity throughout the review process. Inter-reviewer reliability was assessed via Cohen’s Kappa.

### 2.6 Data items and data extraction

Data extraction from the full-text articles was carried out by two authors (A.A. and P.H.), who input the following data items into predefined tables: 1) Bibliographic data (Author, year); 2) Type of study; 3) Type of procedure; 4) Type, donor tissue, and isolation technique of stem cells; 5) Number and demographics of patients; 6) Additional materials, such as bone grafts and membranes, used in the procedure; 7) Clinical, radiographic, histological, and any secondary outcomes; 8) Early and late complications; 9) Follow-up period. Discrepancies in the data extraction process were addressed in consultation with a third author (D.K.).

### 2.7 Risk of bias assessment

Two investigators (P.H. and A.E.A.) independently assessed the quality of the included studies using Cochrane RoB2 tool and ROBINS-I for randomized controlled trials (RCTs) and nonrandomized studies of intervention (NSRIs), respectively (Sterne et al., 2019; Sterne et al., 2016). Inconsistencies were resolved by consulting a third expert author (D.K.), and inter-reviewer agreement was evaluated using Cohen’s Kappa test.

### 2.8 Statistical analysis

A meta-analysis was conducted when at least three controlled studies, either RCTs or CCTs, reported a specific quantitative outcome for similar surgical procedures. Given the variability in outcome measurement scales and units across studies, standardized mean difference (SMD) was selected as the summary effect measure. Specifically, Hedges’ g was used to compute SMDs and their corresponding 95% confidence intervals (CIs), as it offers a bias-corrected and conservative estimate, particularly suitable for studies with small sample sizes (i.e., <20 participants per group). When original studies reported treatment effects using alternative metrics (e.g., mean difference or unadjusted SMD), all estimates were converted to Hedges’ g to ensure a unified effect size metric. Interpretation of effect sizes followed conventional thresholds: small (g = 0.2–0.5), medium (g = 0.5–0.8), and large (g > 0.8). The meta-analysis was performed using the metacont function from the meta package in RStudio (Version, 2024.12.1 + 563, RStudio PBC), with Hedges’ g as the effect size and the restricted maximum likelihood (REML) method for estimating between-study variance (tau^2^). Heterogeneity among studies was quantified using Cochrane’s Q, tau^2^, and Higgins’ I^2^ statistics, with I^2^ values above 75% considered indicative of substantial heterogeneity. Given the heterogeneity in surgical techniques, stem cell types, and follow-up durations across studies, a random-effects model was employed for all primary analyses. Subgroup analyses were also conducted based on follow-up duration, stem cell category used, and surgical procedure. These stratifications allowed for investigation of potential time-, cell-, or procedure-dependent effects of stem-cell-based therapies. A sensitivity analysis was conducted for each meta-analysis by excluding studies classified as having a high overall risk of bias, in order to assess the robustness of the pooled estimates. All statistical analyses and visualizations, including forest plots with subgroup breakdowns, were conducted by a single author (P.H.) using the meta and metafor packages in R. The significance level was set at 0.05 for all analyses, and confidence intervals for tau and I^2^ were computed using the Q-profile method (Balduzzi et al., 2019; Borenstein et al., 2021; Fritz et al., 2012; Schwarzer, 2007).

## 3 Results

### 3.1 Search results

**FIGURE 1:**
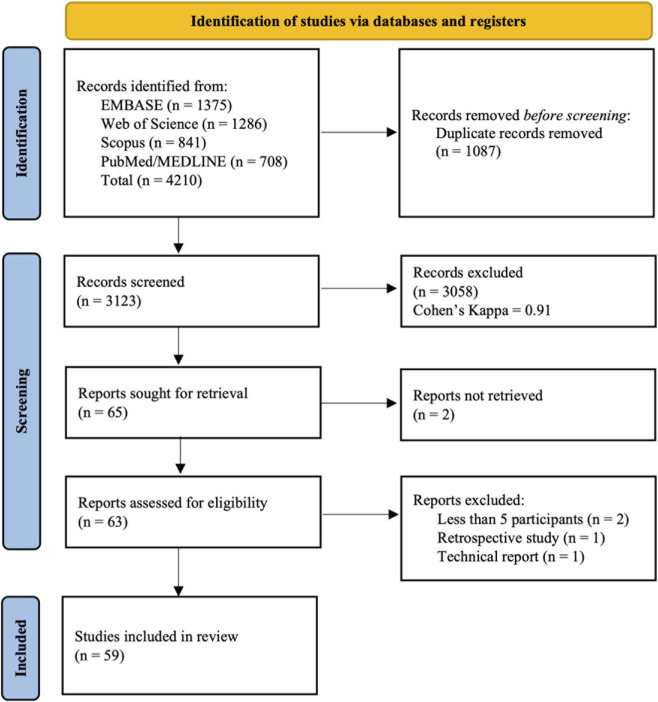
PRISMA flowchart of the review.

As presented in Figure 1, after removing duplicates, 3,123 citations were screened by title and abstract, resulting in 65 studies selected for full-text screening. The inter-reviewer agreement was excellent in title/abstract screening step, with a Cohen’s Kappa value of 0.91 (95% CI: 0.86–0.96). Two of the initially eligible studies could not be retrieved. Among the 62 full-text articles assessed, four were excluded for the following reasons: two had fewer than five participants, one had a retrospective design, and one was a technical note (Supplementary Table S3). Ultimately, 59 studies were included in the review.

### 3.2 Risk of bias assessment

**FIGURE 2:**
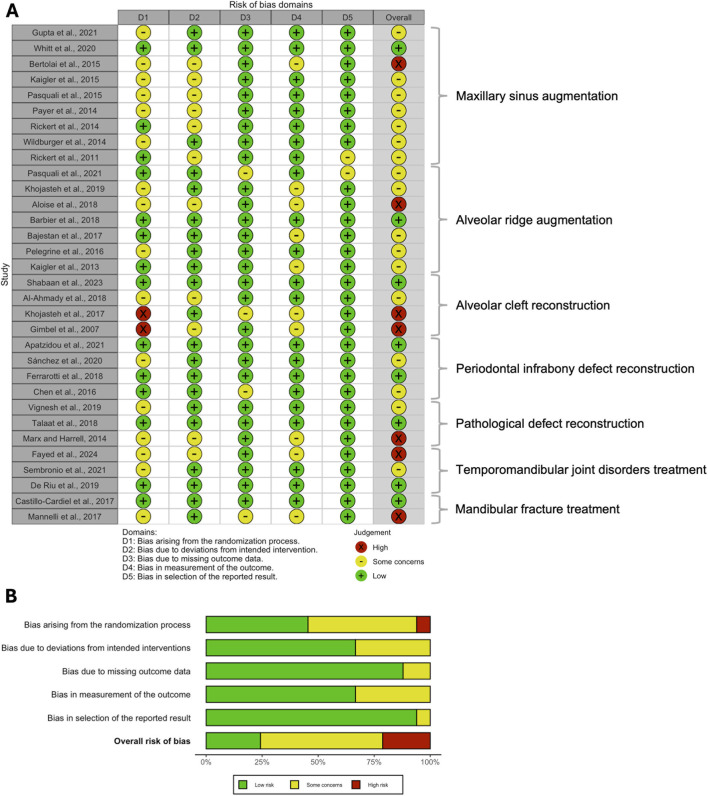
**(A)** Traffic light plot and **(B)** summary bar plot of the risk of bias among the included RCTs, assessed via RoB2 tool.

**FIGURE 3:**
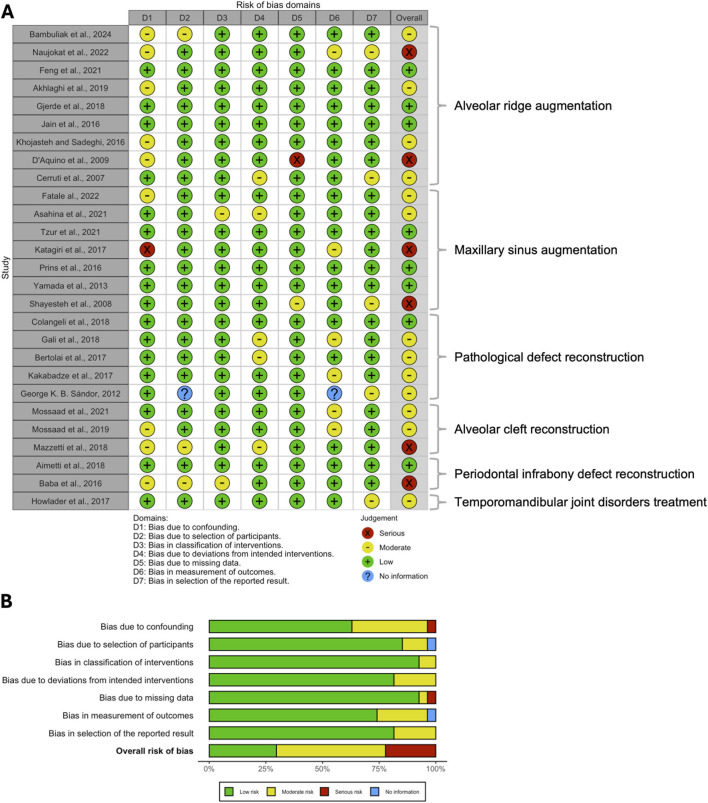
**(A)** Traffic light plot and **(B)** summary bar plot of the risk of bias among the included NRSIs, assessed via ROBINS-I tool.

More than half of the included RCTs were judged to have some concerns regarding the overall risk of bias, while only eight RCTs were appraised as having a low overall risk of bias (Shabaan et al., 2023; Barbier et al., 2018; Castillo-Cardiel et al., 2017; Talaat et al., 2018; Apatzidou et al., 2021; Ferrarotti et al., 2018; Whitt et al., 2020; De Riu et al., 2019) (Figure 2). In contrast, seven RCTs were rated as having a high overall risk of bias (Khojasteh et al., 2017; Gimbel et al., 2007; Aloise et al., 2018; Mannelli et al., 2017; Marx and Harrell, 2014; Bertolai et al., 2015; Fayed et al., 2024). The most common source of bias among the RCTs was related to the randomization process. Similarly, among the non-randomized studies of interventions (NRSIs), nearly half were judged to have a moderate overall risk of bias based on the ROBINS-I tool (Figure 3). Six NRSIs were rated as having a high overall risk of bias (Mazzetti et al., 2018; Naujokat et al., 2022; D'Aquino et al., 2009; Baba et al., 2016; Katagiri et al., 2017; Shayesteh et al., 2008), whereas eight were considered to have a low overall risk of bias (Feng et al., 2021; Gjerde et al., 2018; Jain et al., 2016; Colangeli et al., 2018; Aimetti et al., 2018; Tzur et al., 2021; Prins et al., 2016; Yamada et al., 2013). Inter-reviewer agreement was substantial, with Cohen’s kappa values of 0.84 (95% CI: 0.76–0.92) and 0.81 (95% CI: 0.72–0.90) for the RoB 2 and ROBINS-I tools, respectively.

### 3.3 Characteristics of the included studies

**FIGURE 4:**
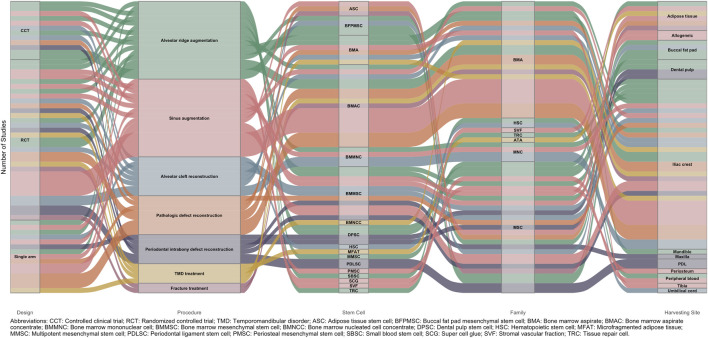
Alluvial plot of the design, procedure, stem cell, stem cell family, and site of harvesting of stem cells among the included studies.

The studies included in this review were published between 2007 and 2024. With regard to study design, the majority were RCTs, accounting for 31 studies. Additionally, 16 were single-arm studies and 11 were CCTs. As illustrated in Figure 4, the included studies evaluated a wide range of regenerative procedures in dentistry and maxillofacial surgery. Alveolar ridge augmentation was assessed in 16 studies (Bambuliak et al., 2024; Naujokat et al., 2022; Feng et al., 2021; Pasquali et al., 2021; Akhlaghi et al., 2019; Khojasteh et al., 2019; Aloise et al., 2018; Barbier et al., 2018; Gjerde et al., 2018; Bajestan et al., 2017; Jain et al., 2016; Khojasteh and Sadeghi, 2016; Pelegrine et al., 2016; Kaigler et al., 2013a; D'Aquino et al., 2009; Cerruti et al., 2007) (Table 2). Likewise, 16 studies focused on maxillary sinus augmentation (Fatale et al., 2022; Asahina et al., 2021; Gupta et al., 2021; Tzur et al., 2021; Whitt et al., 2020; Katagiri et al., 2017; Prins et al., 2016; Bertolai et al., 2015; Kaigler et al., 2015; Pasquali et al., 2015; Payer et al., 2014; Rickert et al., 2014; Wildburger et al., 2014; Yamada et al., 2013; Rickert et al., 2011; Shayesteh et al., 2008) (Table 3). Eight studies evaluated alveolar cleft reconstruction (Shabaan et al., 2023; Mossaad et al., 2021; Mossaad et al., 2019; Al-Ahmady et al., 2018; Mazzetti et al., 2018; Bajestan et al., 2017; Khojasteh et al., 2017; Gimbel et al., 2007) (Table 4),similar to pathologic defect reconstruction that was evaluated in eight studies (Vignesh et al., 2019; Colangeli et al., 2018; Gali et al., 2018; Talaat et al., 2018; Kakabadze et al., 2017; Bertolai et al., 2016; Marx and Harrell, 2014; Sandor, 2012) (Table 5). Periodontal intrabony defect regeneration was investigated in six studies (Apatzidou et al., 2021; Sanchez et al., 2020; Aimetti et al., 2018; Ferrarotti et al., 2018; Baba et al., 2016; Chen et al., 2016) (Table 6). Temporomandibular disorder (TMD) treatment was examined only in four studies (Fayed et al., 2024; De Riu et al., 2019; Howlader et al., 2017; Sembronio et al., 2021) (Table 7). Mandibular fracture treatment was addressed only in two RCTs (Castillo-Cardiel et al., 2017; Mannelli et al., 2017) (Table 8). Only one study assessed two distinct procedures—alveolar cleft reconstruction and alveolar ridge augmentation—across two separate patient cohorts (Bajestan et al., 2017). Figure 4 summarize the study designs, procedures, categories and types of stem cells used, as well as the anatomical locations from which the stem cells were harvested.

**TABLE 2:** Characteristics of the included studies evaluating alveolar ridge augmentation.

Authors, year	Study design	Stem cell	Stem cell location	Stem cell family	Final follow-up	Number of patients in each arm	Male/Female	Age (year) (mean ± SD) [range]	Primary results	Primary conclusion
Bambuliak et al. (2024)	CCT	MMSC	Adipose tissue	MSC	12 months	34	NA	NA	All groups showed favorable shifts in oral markers of bone metabolism over 6–12 months. Bio-Oss + MMSCs + PRP group demonstrated the most pronounced biochemical changes, indicating enhanced bone regeneration	The combination of DBBM + MMSC + PRP appeared most effective in promoting bone remodeling and may be a promising option for alveolar ridge augmentation
						32	NA	NA		
						31	NA	NA		
Naujokat et al. (2022)	CCT	BMAC	Iliac crest	BMA	24 months	33	9/24	60 [31–81]	While implant survival was similar across groups, the BMAC group showed significantly less graft resorption at all time points compared to controls and the DBBM group, with most resorption occurring in the first year	BMAC improved graft stability and may enhance outcomes in complex alveolar ridge reconstruction, despite requiring additional equipment and technical steps
						40	NA	NA		
						40	NA	NA		
Feng et al. (2021)	Single arm	SBSC	Peripheral blood	HSC	6 months	9	5/4	[29–81]	No major adverse effects were reported over 6 months. Mild lab changes were unrelated to the stem cell treatment. SBSC therapy increased cytokine levels associated with tissue repair and improved bone density and stress resistance in patients with severe bone defects	SBSC therapy was safe and showed potential for enhancing bone regeneration and osseointegration in high-risk implant patients
Pasquali et al. (2021)	RCT	BMA	Mandible	BMA	4 months	16	8/8	50.56 ± 5.12	Scintigraphy and histomorphometry revealed significantly higher osteogenic activity and bone formation in the group treated with BMA compared to peripheral blood	Using BMA in block grafting enhanced bone regeneration and osteogenic potential more effectively than conventional grafting with peripheral blood
						16	8/8			
Akhlaghi et al. (2019)	CCT	BFPMSC	Buccal fat pad	MSC	5 months	4	NA	NA	The HAM + BFPMSC group showed significantly greater horizontal bone gain, with slightly improved vertical outcomes, though not statistically significant	Combining HAM with BFPMSC may enhance horizontal bone regeneration while minimizing the need for autogenous bone harvesting and reducing secondary resorption
						5	NA	NA		
Khojasteh et al. (2019)	RCT	BFPMSC	Buccal fat pad	MSC	6 months	7	5/2	[41–65]	CBCT analysis showed similar bone formation between the BFPMSC group and the autologous bone group, with no significant differences across vertical or horizontal defects	BFPMSCs may serve as a viable alternative to autogenous bone for alveolar ridge reconstruction
						7	3/4	[41–65]		
Aloise et al. (2018)	RCT	BMAC	Iliac crest	BMA	8 months	8	NA	NA	Tomographic assessments showed no significant differences between groups. However, histomorphometric analysis revealed greater bone formation in groups treated with BMAC compared to the control group	Adding BMAC to xenograft significantly enhanced bone regeneration, while hyperbaric oxygen therapy offered no additional benefit
						8	NA	NA		
						8	NA	NA		
Barbier et al. (2018)	RCT	DPSC	Dental pulp	MSC	6 months	30	8/22	23 [18–30]	No significant differences in bone density or septum height were observed between groups. Radiologist assessments were consistent	DPSC did not show a measurable effect in reducing bone resorption after third molar extraction in this trial
						30	8/22	23 [18–30]		
Gjerde et al. (2018)	Single arm	BMMSC	Iliac crest	MSC	12 months	11	5/6	[52–79]	BMMSCs combined with calcium phosphate granules led to significant bone regeneration, sufficient for implant placement. Healing was smooth, with no adverse effects, and patients reported high satisfaction	The safety and effectiveness of use of BMMSCs for bone augmentation were confirmed, suggesting it as a promising alternative to traditional grafting methods
Bajestan et al. (2017)	RCT	BMMNC	Iliac crest	MNC	4 months	5	NA	[18–42]	Bone gain was lower in the BMMNC group compared to controls. Wound healing complications were noted, and implant success was limited in BMMNC group, with several patients requiring retreatment using autogenous bone grafts	While stem cell therapy is a safe option for alveolar ridge augmentation, its regenerative capacity remains limited and requires further refinement to match the outcomes of standard grafting methods
						5	NA	[19–54]		
Jain et al. (2016)	CCT	BMMSC	Iliac crest	MSC	6 months	10	NA	[18–35]	Alveolar ridge width was significantly better preserved at grafted sites compared to nongrafted ones	Socket healing with BMMSCs and a collagen membrane effectively maintained alveolar ridge width
						10	NA	[18–35]		
Khojasteh and Sadeghi (2016)	CCT	BFPMSC	Buccal fat pad	MSC	5 months	4	NA	NA	Greater bone width gain, and higher new bone formation were observed in the test group compared to controls	Using BFPMSCs with iliac bone block grafts may enhance bone regeneration and reduce secondary resorption in severely atrophic jaws
						4	NA	NA		
Pelegrine et al. (2016)	RCT	BMAC	Iliac crest	BMA	8 months	4	NA	NA	Radiographic bone gain was comparable between groups, but histomorphometric analysis showed higher mineralized tissue levels in the BMAC group	BMAC may enhance bone mineralization in the anterior maxilla
						4	NA	NA		
Kaigler et al. (2013a)	RCT	TRC	Iliac crest	TRC	12 months	12	6/6	[31–57]	Over 1 year, TRC therapy showed no serious adverse events and led to faster bone regeneration than GBR, with significantly fewer implant-related bone defects	TRC transplantation is a safe and effective approach for accelerating alveolar bone regeneration and reducing the need for secondary grafting, supporting further investigation in craniofacial applications
						12	5/7	[38–63]		
D'Aquino et al. (2009)	Single arm	DPSC	Dental pulp	MSC	3 months	7	1/6	30.29 ± 5.88	Three months after DPSC grafting, patients showed complete periodontal and vertical bone repair, confirmed by clinical and radiographic evaluations. Histology confirmed full bone regeneration	The DPSC + collagen sponge biocomplex effectively restored mandibular bone defects, highlighting its potential for broader tissue and organ regeneration
Cerruti et al. (2007)	Single arm	BMA	Iliac crest	BMA	8 months	32	9/23	[45–75]	CT analysis showed good integration of the scaffolds with the cortical bone	The observed bone healing was attributed to the presence of mesenchymal stem cells within the BMA-enriched grafts

Abbreviations: BFPMSC: buccal fat pad mesenchymal stem cell; BMA: bone marrow aspirate; BMAC: bone marrow aspirate concentrate; BMMSC: bone marrow mesenchymal stem cell; BMMNC: Bone Marrow MonoNuclear Cell; DBBM: deproteinized bovine bone mineral; DPSC: dental pulp stem cell; HAM: human amniotic membrane; HSC: hematopoietic stem cell; MMSC: multipotent mesenchymal stem cell; MNC: MonoNuclear Cell; MSC: mesenchymal stem cell; NA: Not Applicable/Available; PRP: Platelet-Rich Plasma; SBSC: small blood stem cell; TRC: tissue repair cell.

**TABLE 3:** Characteristics of the included studies evaluating maxillary sinus augmentation.

Authors, year	Study design	Stem cell	Stem cell location	Stem cell family	Final follow-up	Number of patients in each arm	Male/Female	Age (year) (mean ± SD) [range]	Primary results	Primary conclusion
Fatale et al. (2022)	CCT	PMSC	Periosteum	MSC	3 months	12	NA	NA	Histomorphometric analysis at 90 days showed a higher percentage of mature bone (Type 1) and lower osteoid tissue (Type 2) in the PMSC group compared to control. This pattern was consistent across both crestal, and lateral sinus lift approaches	Adding PMSCs to the graft material enhanced early bone maturation following sinus lift procedures
						12	NA	NA		
Asahina et al. (2021)	Single arm	BMMSC	Iliac crest	MSC	94 months	8	6/2	54.2	Bone regeneration was achieved in all patients despite individual variation in cell growth and activity. The regenerated bone supported successful implant integration in 27 of 29 sites, with long-term stability over nearly 8 years. No transplant-related adverse events occurred	BMMSC-based grafts with PRP and β-TCP were safe and feasible for treating severe atrophic maxilla through sinus augmentation
Gupta et al. (2021)	RCT	SCG	Peripheral blood	MSC	6 months	20	16/4	36.7	Both groups showed significant postoperative bone height and density gains after sinus lift, with similar healing patterns and implant stability. Membrane perforation occurred in 20% of cases	Stem cell use demonstrated comparable outcomes to blood coagulum in sinus lift procedures, supporting its potential as an alternative grafting approach
						20	16/4	36.7		
Tzur et al. (2021)	Single arm	ASC	Adipose tissue	MSC	6 months	11	9/2	49–65	ASC treatment led to successful bone regeneration without complications or adverse health effects. Notable bone height gains were observed in both sinus augmentation and bone void filling cases	ASC-enhanced graft was safe and effective in generating sufficient bone for implant placement, supporting its potential as a novel autologous grafting material
Whitt et al. (2020)	RCT	BMMSC	Allogeneic	MSC	3.5 months	9	NA	NA	The BMMSC group showed significantly higher vital bone percentage at posterior grafted sites compared to controls. Sinuses were wider posteriorly in both groups, but no anterior–posterior difference in vital bone was observed within groups	Stem cell allografts demonstrated greater osteogenic potential, particularly in wider posterior maxillary sinuses, supporting their use in sinus augmentation procedures
						9	NA	NA		
Katagiri et al. (2017)	CCT	BMMSC	Allogeneic	MSC	6 months	4	2/2	59.8	Bone formation occurred in all cases. While CT density did not differ significantly, histology showed more new bone, particularly lamellar bone, in the BMMSC group, whereas the β-TCP group formed mainly woven bone	BMMSC was safely applied and showed strong osteogenic potential, promoting higher-quality bone regeneration compared to β-TCP.
						2	0/2	70		
Prins et al. (2016)	Single arm	SVF	Adipose tissue	SVF	6 months	10	4/6	56 ± 7 [46–69]	SVF-treated sites showed greater bone and osteoid formation than controls, with no adverse events reported over 3 years	SVF with bone substitutes is safe and may enhance bone regeneration in sinus floor elevation
Bertolai et al. (2015)	RCT	BMAC	Iliac crest	BMA	3 months	20	10/10	55.2	Both groups showed active bone remodeling without inflammation. The BMAC–treated sites had more cellular activity and tighter integration of grafted bone with new bone	Freeze-dried bone engineered with stem cells demonstrated superior histological integration, resembling the behavior of autografts
						20	10/10	55.2		
Kaigler et al. (2015)	RCT	BMMNC	Iliac crest	MNC	4 months	11	NA	NA	Bone volume was similar between groups, but stem cell recipients had higher bone density and quality, especially in severe defects. Greater CD90^+^ cell enrichment correlated with better bone outcomes. No adverse events occurred	CD90^+^ BMMNC–based therapy is safe and improves bone quality in sinus floor reconstruction, with potential for broader craniofacial applications
						12	NA	NA		
Pasquali et al. (2015)	RCT	BMAC	Iliac crest	BMA	6 months	8	NA	55.4 ± 9.2	The BMAC group showed significantly more vital bone and greater resorption of graft material compared to controls, with no difference in non-mineralized tissue	BMAC enhanced bone formation in sinus lift procedures
						8	NA	55.4 ± 9.2		
Payer et al. (2014)	RCT	BMA	Tibia	BMA	6 months	6	3/3	58.2	Tibial and iliac BMA showed similar cell content and bone regeneration. Implants osseointegrated successfully at all sites	Tibial BMA was safe but showed no added regenerative benefit in this small pilot study; its potential may depend on further concentration
						6	3/3	58.2		
Rickert et al. (2014)	RCT	BMAC	Iliac crest	BMA	12 months	12	NA	NA	Three implants failed during osseointegration in the BMAC group, while none failed in the control group. After 1 year, both groups showed similar outcomes in soft tissue health and peri-implant bone levels	Despite early implant failures in the BMAC group, long-term clinical outcomes were comparable between groups
						12	NA	NA		
Wildburger et al. (2014)	RCT	BMAC	Iliac crest	BMA	6 months	7	NA	58 [47–72]	New bone formation and biomaterial resorption were similar between BMAC and control groups at both 3 and 6 months	No significant difference was observed between groups; outcomes may be influenced by the high mineral content of the biomaterial
						7	NA	58 [47–72]		
Yamada et al. (2013)	Single arm	BMMSC	Iliac crest	MSC	6 months	23 (sites)	NA	60.4 [43–74]	BMMSCs led to significant increases in bone height at 3 and 6 months, with no membrane perforation and successful implant outcomes at 1 year	Injectable tissue engineered bone with BMMSCs via osteotome technique is a safe, minimally invasive approach that supports predictable bone regeneration and implant success
Rickert et al. (2011)	RCT	BMAC	Iliac crest	BMA	3 months	12	NA	60.8 ± 5.9 [48–69]	The BMAC group showed significantly more bone formation than the control group. All implants achieved primary stability	BMAC loaded on Bio-Oss effectively promoted new bone formation and may serve as a viable alternative to autografts
						12	NA	60.8 ± 5.9 [48–69]		
Shayesteh et al. (2008)	Single arm	BMMSC	Iliac crest	MSC	13 months	6 (30 sites)	NA	NA	28 out of 30 implants were successful, with no complications reported. Histology showed active bone formation and 41.3% regenerated bone, with no inflammation	BMMSC combined with HA/TCP appears to enhance bone formation in sinus grafting and may be a promising option for implant site development

Abbreviations: ASC: adipose tissue stem cell; BMA: bone marrow aspirate; BMAC: bone marrow aspirate concentrate; BMMSC: bone marrow mesenchymal stem cell; BMMNC: Bone Marrow MonoNuclear Cell; β-TCP: Beta-TriCalcium Phosphate; CT: computed tomography; MSC: mesenchymal stem cell; MNC: MonoNuclear Cell; NA: Not Applicable/Available; PMSC: periostal mesenchymal stem cell; PRP: Platelet-Rich Plasma; RCT: randomized controlled trial; SCG: super cell glue; SVF: stromal vascular fraction.

**TABLE 4:** Characteristics of the included studies evaluating alveolar cleft reconstruction.

Authors, year	Study design	Stem cell	Stem cell location	Stem cell family	Final follow-up	Number of patients in each arm	Male/Female	Age (year) (mean ± SD) [range]	Primary results	Primary conclusion
Shabaan et al. (2023)	RCT	BMAC	Iliac crest	BMA	12 months	18	9/9	[9–11]	The BMAC group showed significantly increased bone volume and density at both 6 and 12 months, while the bone loss ratio significantly declined over the follow-up period	Using a combination of BMAC and iliac cancellous bone in alveolar cleft reconstruction may enhance bone density and volume and reduce graft resorption
						18	10/8	[9–11]		
Mossaad et al. (2021)	Single arm	BMMSC	Iliac crest	MSC	6 months	16	7/9	12.56 ± 1.71	No postoperative complications were observed. Pain and swelling were within acceptable limits. Bone mineral density was comparable between cleft and noncleft sides after 6 months	The BMMSC + PRF graft was successfully used, and DEXA scanning was found to be a simple and effective follow-up tool
Mossaad et al. (2019)	CCT	BMMSC	Iliac crest	MSC	6 months	8	NA	NA	Bone density improved most in the group receiving BMMSC, followed by the nano-hydroxyapatite group. The autogenous iliac crest group showed some graft resorption and donor site complications	BMMSC and nano-hydroxyapatite were more effective and reliable grafting options than autogenous iliac crest
						8	NA	NA		
						8	NA	NA		
Al-Ahmady et al. (2018)	RCT	BMMNC	Iliac crest	MNC	12 months	10	4/6	[8–15]	BMMNC group had fewer donor site complications, better soft tissue healing, and less pain than control group, with higher rates of complete bone union (90% vs. 70%)	The combination of BMMNCs, nanohydroxyapatite, and PRF effectively enhances bone regeneration and offers a promising alternative to conventional grafting
						10	4/6	[8–15]		
Mazzetti et al. (2018)	CCT	HSC	Umbilical cord	HSC	Varying	9	4/5	[0.3–0.5]	No adverse effects were reported in HSC group, and an improved inflammatory response was observed	Safe application and potential benefits of HSC application in soft tissue and bone healing was maintained for 10 years
						9	4/5	[0.3–0.5]		
Bajestan et al. (2017)	RCT	BMMNC	Iliac crest	MNC	4 months	4	NA	NA	Bone gain was lower in the BMMNC group compared to controls. Implant success was lower in the stem cell group. Most complications involved wound healing issues	While stem cell therapy is safe for large alveolar defects, its regenerative capacity remains limited compared to conventional grafting and requires further refinement
						3	NA	NA		
Khojasteh et al. (2017)	RCT	BFPMSC	Buccal fat pad	MSC	6 months	4	NA	NA	Addition of BFPMSC improved results of both iliac and ramus bone graft	Addition of BFPMSC to autogenous grafts improves bone regeneration
						3	NA	NA		
						3	NA	NA		
Gimbel et al. (2007)	RCT	BMA	Iliac crest	BMA	6 months	21	NA	7.8	Traditional iliac crest grafting caused more donor site pain and complications than both tissue engineering and minimally invasive techniques. Tissue engineering had the lowest pain scores and frequency throughout	Using BMA with a resorbable collagen sponge reduced donor site morbidity and pain, offering a less invasive alternative to traditional grafting
						25	NA	8.9		
						23	NA	8.4		

Abbreviations: BFPMSC: buccal fat pad mesenchymal stem cell; BMA: bone marrow aspirate; BMAC: bone marrow aspirate concentrate; BMMSC: bone marrow mesenchymal stem cell; BMMNC: Bone Marrow MonoNuclear Cell; DEXA: Dual-Energy X-ray Absorptiometry; HSC: hematopoietic stem cell; MNC: MonoNuclear Cell; MSC: mesenchymal stem cell; NA: Not Applicable/Available; PRF: Platelet-Rich Fibrin; RCT: randomized controlled trial.

**TABLE 5:** Characteristics of the included studies evaluating pathogenic defect reconstruction.

Authors, year	Study design	Stem cell	Stem cell location	Stem cell family	Final follow-up	Number of patients in each arm	Male/Female	Age (year) (mean ± SD) [range]	Primary results	Primary conclusion
Vignesh et al. (2019)	RCT	BMA	Iliac crest	BMA	6 months	15	8/7	24 ± 9.73	BMA group showed significant bone defect reduction at 3 and 6 months, with less postoperative pain and swelling and no tooth mobility at 3 months	Hydroxyapatite with BMA in a collagen sponge promoted early bone regeneration and faster healing
						15	6/9	28.8 ± 11.88		
Colangeli et al. (2018)	Single arm	BMAC	Iliac crest	BMA	6 months	5	2/3	49.4 ± 7.67	Five patients showed successful bone regeneration with no complications. Newly formed bone had good volume and density	BMAC appears to be a safe and effective option for treating maxillary bone defects
Gali et al. (2018)	Single arm	BMAC	Iliac crest	BMA	6 months	10	5/5	33.7 ± 16.12	All patients showed successful graft integration and new bone formation, with only one minor complication that was resolved with local care	BMAC-coated synthetic hydroxyapatite is effective for regenerating bone in small to moderate mandibular defects
Talaat et al. (2018)	RCT	BMAC	Iliac crest	BMA	12 months	10	6/4	29.81 ± 2.11	Both groups showed significant bone density improvement, but BMAC group had greater density gain and defect size reduction at 6 and 12 months	BMAC with CGF is a safe, cost-effective approach that enhances bone regeneration and density
						10	7/3	30.89 ± 3.27		
Bertolai et al. (2016)	Single arm	BMAC	Iliac crest	BMA	12 months	10	6/4	50 [12–75]	Patients treated with BMAC showed faster healing and 85%–90% ossification of mandibular defects within 12 months, outperforming those without stem cell grafting	Stem cell application significantly enhanced ossification of residual cavities following surgical enucleation
Kakabadze et al. (2017)	CCT	BMMSC	Iliac crest	MSC	5 months	1	1/0	[38–55]	All patients showed uneventful healing with no complications during follow-up after mandibular reconstruction using the graft	Grafts enriched with freeze-dried BMMSC paracrine factors appear promising for reconstructing large mandibular defects post-tumor resection
						3	0/3	[38–55]		
Marx and Harrell (2014)	RCT	BMAC	Iliac crest	BMA	6 months	20	NA	[19–78]	Mature bone regeneration was achieved in all patients with higher CD34^+^ cell counts, while only 40% of the lower-count group reached this outcome. Bone density and trabecular bone area were also significantly higher in the high-CD34^+^ group	CD34^+^ cells play a key role in bone regeneration, with sufficient concentrations in BMAC being critical for successful outcomes in stem cell-based grafts
						20	NA	[19–78]		
Sandor (2012)	Single arm	ASC	Adipose tissue	MSC	NA	23	NA	NA	Out of 23 ASC + scaffold reconstructions combined with rhBMP-2, 20 were successful. Failures included one infection and two cases of insufficient bone formation	Although ASC-aided jaw reconstruction is currently time-consuming and costly, early results are promising and may lead to wider clinical use with future technological improvements

Abbreviations: ASC: adipose tissue stem cell; BMA: bone marrow aspirate; BMAC: bone marrow aspirate concentrate; BMMSC: bone marrow mesenchymal stem cell; CCT: controlled clinical trial; CGF: concentrated growth factor; MSC: mesenchymal stem cell; NA: Not Applicable/Available; RCT: randomized controlled trial; rhBMP-2: recombinant Human Bone Morphogenetic Protein-2.

**TABLE 6:** Characteristics of the included studies evaluating periodontal intrabony defect reconstruction.

Authors, year	Study design	Stem cell	Stem cell location	Stem cell family	Final follow-up	Number of patients in each arm	Male/Female	Age (year) (mean ± SD) [range]	Primary results	Primary conclusion
Apatzidou et al. (2021)	RCT	BMMSC	Maxilla	MSC	12 months	9	3/6	49.7 ± 4.8	All treatment groups showed significant clinical improvements with no adverse events. While clinical outcomes were comparable, BMMSC and access flap surgery groups demonstrated greater radiographic bone fill than collagen scaffold without stem cell group	BMMSC-based therapies enhanced bone regeneration radiographically, though all approaches effectively promoted periodontal healing through activation of innate tissue repair
						10	5/5	49.9 ± 8.7		
						8	2/6	54.9 ± 7.2		
Sanchez et al. (2020)	RCT	PDLSC	PDL	MSC	12 months	10	7/3	48.8 ± 10.6	The PDLSC group showed slightly greater CAL gain and PPD reduction than controls, but differences were not statistically significant. No serious adverse events occurred	PDLSCs combined with xenograft were safe and well-tolerated but did not show clear added benefit over xenograft alone in treating intra-bony lesions
						10	7/3	57.5 ± 7.9		
Aimetti et al. (2018)	Single arm	DPSC	Dental pulp	MSC	12 months	11	6/5	51.2 ± 6.1	At year 1, there was a mean CAL gain of 4.7 mm and stable gingival margins, with 63.6% of sites achieving complete pocket closure. Radiographs showed an average bone fill of 3.6 mm	The treatment resulted in significant clinical and radiographic improvements, including substantial attachment gain and bone regeneration
Ferrarotti et al. (2018)	RCT	DPSC	Dental pulp	MSC	12 months	15	8/7	51.9 ± 8.4	The DPSC group showed significantly greater improvements in PD reduction, CAL gain, and bone fill compared to controls. A higher percentage of sites achieved favorable clinical thresholds	DPSC application significantly enhanced periodontal regeneration outcomes after 1 year
						14	6/8	49.4 ± 9.3		
Baba et al. (2016)	CCT	BMMSC	Iliac crest	MSC	36 months	10	3/7	48.4	Over 36 months, patients showed significant improvements in CAL, PD, and linear bone growth, with no safety concerns related to the MSC therapy	BMMSC-based therapy with PRP and a 3D scaffold appears to be a safe and effective regenerative approach for treating periodontitis
						10	3/7	48.4		
Chen et al. (2016)	RCT	PDLSC	PDL	MSC	12 months	20	2/18	26.05 ± 4.44	Both groups showed significant bone height improvement over time, with no adverse events linked to PDLSC treatment. However, no significant difference was found between the cell and control groups	PDLSC therapy for periodontal defects is safe, but its added benefit remains uncertain and requires confirmation through larger, multicenter trials
						21	6/15	30.04 ± 7.90		

Abbreviations: BMMSC: bone marrow mesenchymal stem cell; CAL: clinical attachment level; CCT: controlled clinical trial; DPSC: dental pulp stem cell; MSC: mesenchymal stem cell; PD: pocket depth; PDLSC: periodontal ligament stem cell; PDL: periodontal ligament; PRP: Platelet-Rich Plasma; RCT: randomized controlled trial.

**TABLE 7:** Characteristics of the included studies evaluating temporomandibular joint disorders (TMD) treatment.

Authors, year	Study design	Stem cell	Stem cell location	Stem cell family	Final follow-up	Number of patients in each arm	Male/Female	Age (year) (mean ± SD) [range]	Primary results	Primary conclusion
Fayed et al. (2024)	RCT	BMAC	Iliac crest	BMA	18 months	12	2/10	44.3 ± 8.3	The BMAC group showed sustained joint repair and symptom improvement at 12 and 18 months, unlike the HA group, which experienced relapse	BMAC may reverse TMJ osteoarthritis progression
						12	4/8	43.1 ± 9.4		
Sembronio et al. (2021)	RCT	MFAT	Adipose tissue	ATA	6 months	20	NA	43.3 ± 21.4	Pain scores were lower in MFAT group compared to control at 10-day and 1-month post-operative. Also, mouth opening was higher in MFAT group at 6 months	MFAT injection in conjunction with arthrocentesis results in lower pain and higher mouth opening in internal derangement and osteoarthritis of TMJ.
						20	NA	50.7 ± 17.4		
De Riu et al. (2019)	RCT	BMNCC	Iliac crest	BMA	12 months	15	0/15	48.2 ± 10.2 [35–67]	Both groups improved clinically, but the BMNCC group showed significantly better outcomes in pain relief, chewing efficiency, and mouth opening at 6 and 12 months. No cartilage regeneration was seen on MRI.	Intra-articular BMNCC injection led to superior clinical improvement in TMD patients, suggesting promising potential for this treatment
						15	1/14	44.5 ± 12.6 [33–61]		
Howlader et al. (2017)	Single arm	BMA	Iliac crest	BMA	12 months	7	5/2	9.71 ± 3.3 [5–14]	Significant improvements were observed in mouth opening, jaw movement, and TMJ function over 1 year, with a high success score reported	HA/Collagen bio-scaffold with BMA is a safe, effective, and affordable option for mandibular condyle reconstruction, especially in growing patients

Abbreviations: ATA: adipose tissue aspirate; BMA: bone marrow aspirate; BMAC: bone marrow aspirate concentrate; BMNCC: bone marrow nucleated cell concentrate; MFAT: MicroFragmented Adipose Tissue; MRI: magnetic resonance imaging; RCT: randomized controlled trial; TMJ: temporomandibular joint.

**TABLE 8:** Characteristics of the included studies evaluating mandibular fracture treatment.

Authors, year	Study design	Stem cell	Stem cell location	Stem cell family	Final follow-up	Number of patients in each arm	Male/Female	Age (year) (mean ± SD) [range]	Primary results	Primary conclusion
Castillo-Cardiel et al. (2017)	RCT	ASC	Adipose tissue	MSC	3 months	10	10/0	31.2 ± 6.3	Bone quality was significantly higher in the ASC group compared to controls at both 4 and 12 weeks, with a more pronounced difference at 12 weeks	While early ossification was similar, ASCs significantly enhanced bone regeneration by 12 weeks compared to standard mandibular fracture reduction alone
						10	10/0	29.7 ± 7.2		
Mannelli et al. (2017)	RCT	BMAC	Iliac crest	BMA	12 months	17	5/12	[66–85]	Among 35 patients, complications were fewer in the BMAC group, with lower rates of non-union and infection compared to controls	Using BMAC in managing atrophic mandibular fractures is safe, reduces complication rates, and supports effective recovery even in medically complex patients

Abbreviations: ASC: adipose tissue stem cell; BMA: bone marrow aspirate; BMAC: bone marrow aspirate concentrate; MSC: mesenchymal stem cell; RCT: randomized controlled trial.

### 3.4 Results of the individual studies according to the type of procedure

#### 3.4.1 Alveolar ridge augmentation

The clinical outcomes indicated that various regenerative strategies, including bone fillers and stem cell-based approaches, led to high implant survival rates and effective bone regeneration. In several studies, there were no significant complications, with patients exhibiting good bone healing and minimal adverse events (Naujokat et al., 2022; Barbier et al., 2018). Additionally, soft tissue healing was generally good, with fewer complications and faster recovery in the test groups. In particular, the bone formation in patients treated with stem cells or biological materials was more pronounced (Kaigler et al., 2013a; Akhlaghi et al., 2019). Furthermore, the test groups showed less postoperative pain and swelling, suggesting that these advanced techniques may lead to a better patient experience during recovery (Aloise et al., 2018).

From a histological standpoint, the studies consistently showed superior bone formation in the test groups. Bone biopsies showed increased mineralized tissue and active osteoblast activity, which is indicative of successful bone regeneration (Kaigler et al., 2013a; Naujokat et al., 2022). The presence of newly formed bone with lamellar structure and well-organized osteoblasts was particularly noted in groups treated with bone marrow aspirate concentrate (BMAC) and growth factors (Aloise et al., 2018; Feng et al., 2021). Although one study observed minimal or no significant histological differences between groups, the overall trend pointed toward better bone healing and tissue integration in the experimental groups (Barbier et al., 2018).

Radiographically, the test groups demonstrated significant improvements in bone volume, height, and width compared to control groups. Bone resorption rates were lower, and increased bone density was noted at various time points, particularly at 6 months post-procedure (Naujokat et al., 2022). Additionally, many studies reported an increase in bone thickness, with CT scans showing well-integrated scaffolds and improved graft stability (Cerruti et al., 2007; D'Aquino et al., 2009). However, while these regenerative approaches resulted in favorable radiographic outcomes, some studies did not observe significant bone formation in all participants, particularly in cases where less effective graft materials or inadequate stem cell concentrations were used (Barbier et al., 2018).

#### 3.4.2 Sinus augmentation

The use of stem cells in sinus augmentation procedures has shown promising results across various studies, particularly in terms of bone regeneration and implant stability. Clinically, several studies reported a high success rate of implant placement following sinus grafting with stem cell-enriched biomaterials. For instance, Gupta et al. and Yamada et al. both observed a 100% success rate in their test groups, with faster healing times and reduced postoperative complications (Yamada et al., 2013; Gupta et al., 2021). Similarly, Shayesteh et al. reported a 93% success rate, demonstrating that the incorporation of MSCs into bone grafts could enhance implant stability (Shayesteh et al., 2008). However, other studies did not report notable clinical outcomes but instead reported on histological and radiographic assessments (Fatale et al., 2022).

Histological analyses have consistently demonstrated improved bone regeneration in test groups where stem cells were used. Katagiri et al. found that lamellar bone formation was significantly higher in the MSC group, whereas control groups primarily exhibited woven bone (Katagiri et al., 2017). Similarly, Whitt et al. and Rickert et al. reported greater percentages of newly formed vital bone in their test groups (50.12% and 17.7%, respectively) compared to their controls (Whitt et al., 2020; Rickert et al., 2011). Fatale et al. also found a higher percentage of type I bone (44.45%) in MSC-treated sites versus 27.24% in the control group (Fatale et al., 2022). Moreover, some groups have observed enhanced bone regeneration in test groups, confirming the osteogenic potential of stem cells when combined with biomaterials like DBBM (Wildburger et al., 2014; Bertolai et al., 2015).

Radiographic evaluations further supported the superior performance of stem cell-enhanced grafts in promoting bone formation and stability. Several studies found significantly less bone resorption in test groups compared to controls (Gupta et al., 2021; Rickert et al., 2011). Increased bone height and density were frequently reported, with Yamada et al. documenting notable postoperative improvements in residual bone height. Likewise, Prins et al. and Payer et al. observed enhanced vertical bone height and implant stability in stem cell-treated groups (Prins et al., 2016; Payer et al., 2014). Interestingly, Katagiri et al. found no significant differences in radiographic HU between test and control groups, suggesting that while stem cells accelerate bone formation, their effect on bone mineral density may require further investigation (Katagiri et al., 2017).

#### 3.4.3 Alveolar cleft reconstruction

Clinical outcomes consistently showed significant improvements in soft tissue healing, with several studies also reporting reduced post-operative pain and inflammation when using stem cells (Shabaan et al., 2023; Mossaad et al., 2021; Gimbel et al., 2007; Al-Ahmady et al., 2018; Mazzetti et al., 2018). Histological findings revealed increased bone density in groups treated with stem cells (Khojasteh et al., 2017). The presence of lamellar bone with active osteoblastic rims and minimal inflammation supports the hypothesis that stem cell-based methods enhance the formation of high-quality bone. However, some studies did not observe significant bone development, highlighting the need to evaluate additional factors influencing osteogenesis (Mazzetti et al., 2018).

Radiographic assessments were consistent with clinical and histological findings, demonstrating higher bone fill rates in the test groups compared to controls. Bone density measurements via computed tomography (CT) scans showed superior outcomes in regeneration groups employing stem cells, with reduced postoperative bone resorption and improved graft integration at the recipient site (Khojasteh et al., 2017; Mossaad et al., 2019). These results suggest that biomaterials and stem cell-based approaches may enhance long-term stability of the alveolar defect, although some studies did not confirm significant bone formation in all cases (Mazzetti et al., 2018).

#### 3.4.4 Pathogenic defect reconstruction

Clinically, most studies reported high success rates and enhanced bone regeneration in stem cell-treated groups. For instance, in Kakabadze et al.’s study, one of the only two studies that did not use BMA and used MSC to treat pathogenic defects, observed a 100% success rate with significantly higher bone density in the test group (Kakabadze et al., 2017). Similarly, Marx and Harrell found that patients receiving BMA had a 100% success rate compared to 40% in control group. Vignesh et al. and Gali et al. both reported faster bone healing and improved overlying mucosal healing in BMA groups, further supporting the positive impact of stem cell therapy on defect reconstruction (Vignesh et al., 2019; Gali et al., 2018). Additionally, Bertolai et al. and Colangeli et al. confirmed successful bone regeneration in all patients treated with BMA, with no significant complications reported postoperatively (Bertolai et al., 2016; Colangeli et al., 2018).

Histologically, studies demonstrated that stem cell-enhanced grafts contributed to better-organized bone formation and vascularization. For example, George K. B. Sándor and Bertolai et al. observed well-organized bone regeneration with abundant vascular structures, suggesting improved integration of the newly formed bone (Bertolai et al., 2016; Sandor, 2012). Vignesh et al. identified the presence of bone marrow hematopoietic and mesenchymal elements in the test group, further confirming the osteogenic and angiogenic potential of stem cell therapy. Moreover, Kakabadze found significant new bone formation in MSC-treated sites, whereas the control group showed limited bone regeneration (Kakabadze et al., 2017). In contrast, Talaat et al. did not report specific histological findings, highlighting a gap in data that limits direct comparisons across all studies (Talaat et al., 2018).

Radiographic analyses consistently supported the clinical and histological findings, indicating increased bone density and volume preservation in stem cell-treated groups. Marx and Harrell reported that MSC therapy exhibited a higher bone density compared to control, which had a lower success rate (Marx and Harrell, 2014). Similarly, Vignesh et al. and Talaat et al. observed significant reductions in defect volume and increased bone density in test groups (Vignesh et al., 2019; Talaat et al., 2018). Colangeli et al. reported a mean bone density of 850 Hounsfield unit (HU) at 6 months postoperatively, further demonstrating the efficacy of BMA-based therapies in maintaining bone structure (Colangeli et al., 2018). Bertolai et al. and Kakabadze et al. also documented superior bone volume maintenance in stem cell-treated sites compared to controls (Kakabadze et al., 2017; Bertolai et al., 2016). However, Gali et al. noted some bone resorption at 3 months, suggesting that while stem cell therapy promotes initial bone regeneration, long-term stability may still require further investigation (Gali et al., 2018).

#### 3.4.5 Periodontal intrabony defect reconstruction

Clinically, to treat intrabony defects, studies have reported significant reductions in probing depth (PD) and gains in CAL, albeit with variability in success rates. Apatzidou et al. found a 55.6% success rate in the stem cell group, defined as cases achieving at least 3 mm of CAL gain (Apatzidou et al., 2021), while Sánchez et al. observed a mean CAL gain of 1.44 mm, though the difference compared to the control group was not statistically significant (Sanchez et al., 2020). Ferrarotti et al. and Aimetti et al. reported greater improvements, with PD reductions of 4.9 ± 1.4 mm and 5.0 ± 1.3 mm, respectively, and CAL gains of 4.7 ± 1.6 mm in the test groups, indicating that MSC-based treatments may enhance periodontal healing (Ferrarotti et al., 2018; Aimetti et al., 2018). Baba et al. further confirmed significant gains in PD, CAL, and linear bone growth (LBG), suggesting a consistent clinical advantage in MSC-treated defects (Baba et al., 2016).

Histological analyses supported these clinical findings, demonstrating improved bone regeneration and osteogenic activity in MSC-treated defects. Apatzidou et al. reported enhanced bone regeneration in the stem cell group compared to controls (Apatzidou et al., 2021), while Chen et al. observed a more integrated graft structure and reduced connective tissue encapsulation in the test group, indicating superior osseointegration (Chen et al., 2016). Baba et al. identified high alkaline phosphatase (ALP) activity and elevated osteogenic markers, reinforcing the hypothesis that MSC-based therapy promotes active bone remodeling and regeneration. However, the lack of histological data in some studies (Aimetti et al., 2018; Ferrarotti et al., 2018; Sanchez et al., 2020) limits a comprehensive comparison across trials.

Radiographic outcomes further validated these findings, with most studies reporting increased bone fill and defect resolution. Apatzidou et al. documented bone fill at 12 months in both test and control groups (Apatzidou et al., 2021), while Ferrarotti et al. and Aimetti et al. reported mean radiographic bone fills of 3.9 ± 1.5 mm and 3.6 ± 1.9 mm, respectively, in MSC-treated sites, suggesting substantial periodontal regeneration (Ferrarotti et al., 2018; Aimetti et al., 2018). Chen et al. found that defects treated with periodontal ligament stem cells (PDLSCs) and deproteinized bovine bone mineral (DBBM) exhibited significantly greater bone fill compared to control sites (Chen et al., 2016). Baba et al. further confirmed that the test group showed superior linear bone growth compared to controls (Baba et al., 2016). However, Sanchez et al. did not find significant differences in radiographic bone fill between groups, indicating potential variability in outcomes depending on patient-specific factors and treatment protocols (Sanchez et al., 2020).

#### 3.4.6 TMD treatment

Clinically, all four included studies consistently reported significant reductions in pain and improved mandibular mobility in patients receiving BMA or adiposte tissue aspirate (ATA)-based treatments. De Riu et al. observed a significantly greater reduction in pain in the test group compared to the control, suggesting a potential analgesic effect of BMA (De Riu et al., 2019). Similarly, Fayed et al. found that, after 1 week and 1 month, the test group showed significantly greater improvements in mandibular function and reduced discomfort relative to the control group (Fayed et al., 2024). Additionally, Howlader et al. documented a remarkable increase in mouth opening—from 4.14 mm to 34.57 mm post-treatment—highlighting the promise of stem cell therapy in restoring joint mobility and alleviating TMD symptoms (Howlader et al., 2017). The only study in this review that investigated microfragmented adipose tissue (MFAT) evaluated its use in combination with arthrocentesis and found greater improvements in both pain and mouth opening compared to arthrocentesis with hyaluronic acid injection alone (Sembronio et al., 2021).

Although histological data were absent in these studies, radiographic evaluations provided substantial evidence of bone regeneration and joint remodeling. De Riu et al. reported significantly greater bone formation in the BMA-treated group, supporting the role of stem cells in regenerating TMJ structures (De Riu et al., 2019). Fayed et al., through an 18-month postoperative radiographic evaluation, observed improved joint morphology in the test group, reinforcing the regenerative potential of BMA (Fayed et al., 2024). Similarly, Howlader et al. used CT and magnetic resonance imaging (MRI) to assess joint integrity, revealing enhanced bone structure and reduced joint degeneration in the BMA-treated group (Howlader et al., 2017).

#### 3.4.7 Mandibular fracture

Clinically, Mannelli et al. observed after 3 months stable functional outcomes in all patients who were treated for fractures of the atrophic mandible, suggesting that bone marrow aspirate (BMA)-based therapy may contribute to enhanced fracture stability and recovery (Mannelli et al., 2017). Similarly, Castillo-Cardiel et al. reported a reduced recovery time in mandibular fracture cases treated with MSC-based treatments, with patients experiencing earlier functional improvements compared to the control group (Castillo-Cardiel et al., 2017).

Histological analyses were not conducted in these studies. Radiographically, both studies demonstrated favorable outcomes with stem cell therapy. Mannelli et al. reported stable long-term functional integrity in all patients, indicating that stem cell treatment may promote sustained bone healing (Mannelli et al., 2017). Castillo-Cardiel et al. conducted radiographic assessments at multiple time points, finding that by week four, the test group exhibited better callus formation and bone consolidation compared to controls (Castillo-Cardiel et al., 2017).

### 3.5 Meta-analysis

#### 3.5.1 Periodontal clinical attachment level (CAL) gain

**FIGURE 5:**
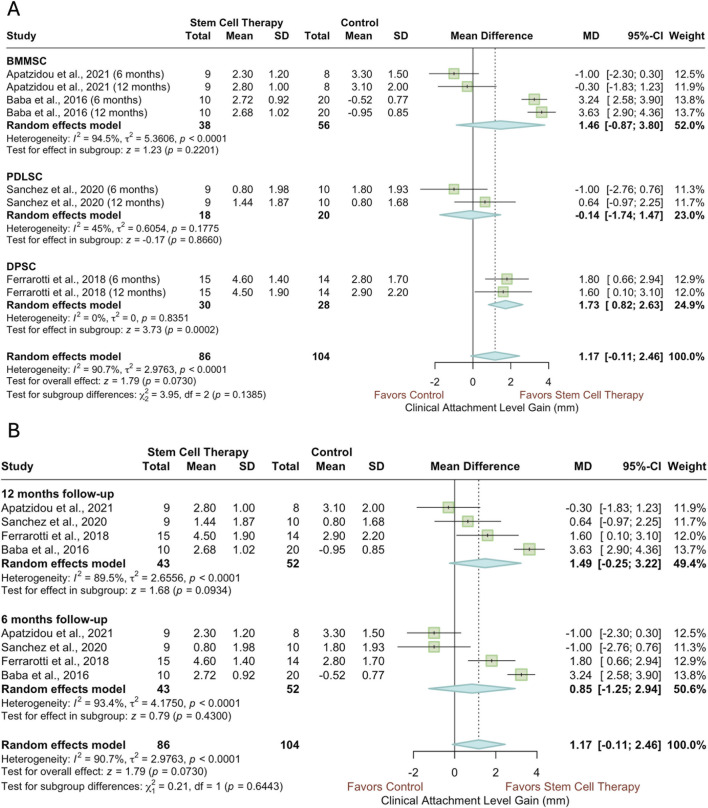
Forest plots of CAL gain, subgrouped according to **(A)** type of stem cell and **(B)** follow-up period.

Four studies, three RCTs and one CCT, each reporting outcomes at both six and 12 months, were included in the meta-analysis of CAL gain (Figure 5). As shown in Figure 5A, a subgroup analysis based on the type of stem cell used revealed that the dental pulp stem cell (DPSC) subgroup demonstrated a significant CAL gain (SMD = 1.73, 95% CI = 0.82 to 2.63, *p* = 0.0002). In contrast, no significant effects were observed in the PDLSC and bone marrow mesenchymal stem cell (BMMSC) subgroups (PDLSC: SMD = −0.14, 95% CI = −1.74 to 1.47, *p* = 0.8660; BMMSC: SMD = 1.46, 95% CI = −0.87 to 3.80, *p* = 0.2201). Also, a significant heterogeneity in the results of studies employing BMMSC was noted (*I* ^2^ = 94.5%, *p* < 0.0001). When stratified by follow-up duration (Figure 5B), substantial heterogeneity was observed within both subgroups as well as overall (*p* < 0.0001). No significant treatment effect of stem cell application was detected in either subgroup or in the overall estimate (6 months: SMD = 0.85, 95% CI = −1.25 to 2.94, *p* = 0.4300; 12 months: SMD = 1.49, 95% CI = −0.25 to 3.22, *p* = 0.0934; overall: SMD = 1.17, 95% CI = −0.11 to 2.46, *p* = 0.0730). As shown in Supplementary Figure S1, the sensitivity analysis excluding Baba et al.’s study did not alter the findings of this analysis (Baba et al., 2016).

#### 3.5.2 Quality of the regenerated bone

**FIGURE 6:**
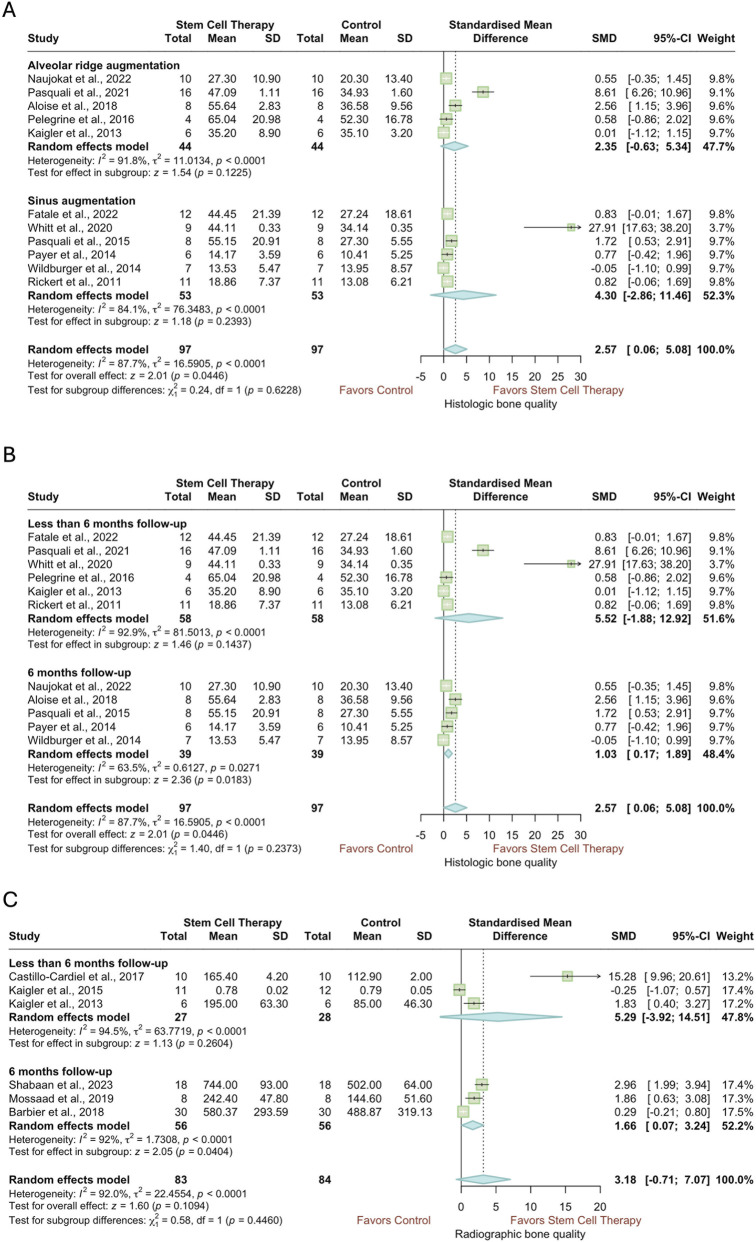
Forest plots of the quality of regenerated bone according to histology, subgrouped according to **(A)** type of procedure, **(B)** follow-up period. **(C)** Forest plots of the quality of regenerated bone according to radiography, subgrouped according to follow-up period.

The quality of regenerated bone was evaluated using both histological and radiographic assessments in the included studies (Figure 6). A subgroup analysis based on surgical procedure, limited to studies using histology, showed that stem cell therapy did not significantly improve bone quality in either alveolar ridge augmentation (SMD = 2.35, 95% CI = −0.63 to 5.34, *p* = 0.1225) or sinus augmentation (SMD = 4.30, 95% CI = −2.86 to 11.46, *p* = 0.2393). However, the overall effect across all procedures was statistically significant (SMD = 2.57, 95% CI = 0.06 to 5.08, *p* = 0.0446), indicating a very large effect size (Figure 6A). Substantial and statistically significant heterogeneity was observed within both subgroups and overall (*p* < 0.0001). When the same studies were stratified by follow-up duration, the effect was significant, yet small, only among those with a 6-month follow-up (SMD = 1.03, 95% CI = 0.17 to 1.89, *p* = 0.0271). In contrast, studies with follow-up periods shorter than 6 months did not demonstrate a significant effect (SMD = 5.52, 95% CI = −1.88 to 12.92, *p* = 0.1437) (Figure 6B). Heterogeneity remained considerable and statistically significant in both follow-up subgroups. Excluding the two studies with a high overall risk of bias (Naujokat et al., 2022; Aloise et al., 2018) in the sensitivity analysis changed the statistical significance of the overall finding, as well as the effect observed in the 6-month follow-up subgroup (Supplementary Figure S2). A similar trend was observed in a separate meta-analysis including studies using radiography to evaluate regenerated bone’s quality (Figure 6C). The overall effect of stem cell therapy was insignificant (SMD = 3.18, 95% CI: -0.71 to 7.07, *p* = 0.1094); however, the effect was significant in 6 months subgroup (SMD = 1.66, 95% CI = 0.07 to 3.24, *p* = 0.0404). Substantial and significant heterogeneity was observed among the studies of both subgroups, as well as overall (*p* < 0.0001). Since all studies included in this analysis were rated as having either low risk or some concerns in the overall risk of bias, no sensitivity analysis was performed.

#### 3.5.3 Quantity of the regenerated bone

**FIGURE 7:**
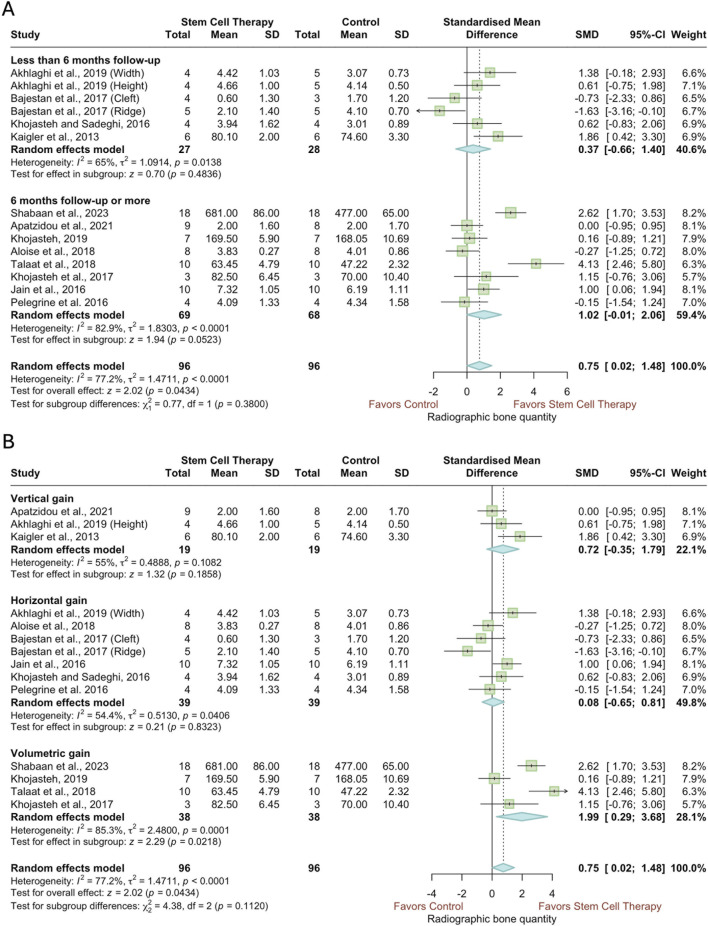
Forest plots of the quantity of regenerated bone according to radiography, subgrouped according to **(A)** follow-up period, **(B)** dimension of change.

Stratified according to follow-up period (Figure 7A), studies evaluating regenerated bone quantity with radiography demonstrated significant heterogeneity (less than 6 months of follow-up: *I* ^2^ = 65%, *p* = 0.0138; 6 months of follow-up: *I* ^2^ = 82.9%, *p* < 0.0001; overall: *I* ^2^ = 77.2%, *p* < 0.0001). Even though the effect was not significant in either of the less than 6 months or 6 months subgroups (less than 6 months of follow-up: SMD = 0.37, 95% CI: -0.66 to 1.40, *p* = 0.4836; 6 months of follow-up: SMD = 1.02, 95% CI: -0.01 to 2.06, *p* = 0.0523), the overall effect of stem cell therapy was significant (SMD = 0.75, 95% CI = 0.02 to 1.48, *p* = 0.0434). As presented in Figure 7B, when the studies were stratified according to the dimension of measuring the regenerated bone, in both linear subgroups—vertical and horizontal—had insignificant effect sizes (vertical: SMD = 0.72, 95% CI = −0.35 to 1.79, *p* = 0.1858; horizontal: SMD = 0.08, 95% CI = −0.65 to 0.81, *p* = 0.8323). However, a very large effect size was observed in the three-dimensional subgroup (SMD = 1.99, 95% CI: 0.29 to 3.68, *p* = 0.0218), in spite of having substantial and significant heterogeneity (*I* ^2^ = 85.3%, *p* = 0.0001). When the two studies with high risk of bias (Khojasteh et al., 2017; Aloise et al., 2018) were excluded in the sensitivity analysis, the overall effect became insignificant, while the significant effect observed in the volumetric gain subgroup remained unchanged (Supplementary Figure S3).

## 4 Discussion

This systematic review and meta-analysis provides a comprehensive evaluation of current clinical evidence regarding the use of stem cell-based therapies for oral and craniofacial bone regeneration. By synthesizing data from 59 prospective studies encompassing diverse clinical indications—including alveolar cleft repair, ridge augmentation, sinus augmentation, periodontal intrabony defects, and TMJ disorders—this review offers a comprehensive perspective on the therapeutic applications of stem cells across the craniofacial region. The majority of included studies reported favorable outcomes, demonstrating that stem cell-based interventions can enhance both the quality and quantity of regenerated bone compared to conventional methods. Notably, pooled meta-analytic findings further corroborated these benefits, with stem cell therapies being consistently associated with superior volumetric bone regeneration and improved histological bone quality. These results underscore the growing potential of stem cell-centered strategies to address the limitations of traditional grafting approaches in periodontal, alveolar, and craniofacial reconstruction. In a recent systematic review, Hung et al. included clinical studies published between 2013 and 2023 evaluating the effect of stem cell therapy on the regenerative outcomes of procedures on maxillofacial bone tissues (Hung et al., 2024). They included seven studies, all of those meeting the eligibility criteria of the present study are included in this review, and highlighted the efficacy, safety, and effectiveness of stem cell-based approaches (Hung et al., 2024). Similarly, Dipalma et al. conducted a systematic review encompassing 12 studies, including clinical and laboratory studies, and concluded that although MSC-based therapies represent a promising frontier in maxillofacial bone regeneration, the broad variability in study methodologies, stem cell preparation protocols, and outcome measures highlights the importance of establishing more standardized approaches (Dipalma et al., 2025). It is noteworthy that despite other systematic reviews being recently conducted, the number of studies employing a meta-analysis to quantify the effect of stem cell therapy on craniofacial bone regeneration are scarce. To our knowledge, the only meta-analysis which has been conducted in this area was conducted by Eini et al. and they reported that stem cells combined with scaffolds improve bone regeneration (Eini et al., 2023). In addition to multiple meta-analyses for different outcome measures, the current study also includes various subgroup analyses aimed at addressing the sources of heterogeneity frequently observed among the studies. However, true heterogeneity in designs and materials, along with the scarcity of certain categories of stem cells, study designs, or follow-up periods, limited the possibility of further subgroup analyses, as these often resulted in single-study subgroups, which the Cochrane Handbook for Systematic Reviews explicitly discourages (Deeks et al., 2023).

Tissue engineering for bone regeneration is founded on three fundamental pillars: stem cells, scaffolds, and biological agents (Hao et al., 2021). A substantial body of translational, *in vitro*, and preclinical animal research has elucidated the mechanisms through which stem cell-based therapies contribute to bone formation and regeneration (Arvidson et al., 2011; Garg et al., 2017; Baniameri et al., 2025). These studies consistently demonstrate the osteogenic and immunomodulatory potential of stem cells, particularly when delivered within an optimized microenvironment. Concurrently, extensive research is underway to evaluate approaches aimed to enhance the osteoinductive and osteoconductive properties of scaffolds and biological agents, aiming to create more favorable conditions for stem cell survival, differentiation, and integration. Together, these three components form the core of regenerative strategies designed to mimic and support native bone healing processes (Nokhbatolfoghahaei et al., 2025; Mohaghegh et al., 2024).

Safety—often a central concern in stem cell-based regenerative interventions—was consistently reported across the included studies (Asahina et al., 2021; Katagiri et al., 2017; Howlader et al., 2017; Bajestan et al., 2017; Feng et al., 2021; Gjerde et al., 2018; Castillo-Cardiel et al., 2017; Sanchez et al., 2020). No serious complications attributable to stem cell application were observed beyond common postoperative effects such as pain, swelling, or minor bleeding. These findings are in agreement with earlier reviews that have supported the favorable safety profile of stem cell therapies in maxillofacial applications (Nguyen-Thi et al., 2023; Liu et al., 2022; Tatullo et al., 2015). This is consistent with a large body of research demonstrating that stem cell-based therapies are generally safe across a wide range of medical fields, including not only oral and craniofacial procedures but also orthopedic and other surgical applications (Trounson and McDonald, 2015). In addition to its safety, a substantial body of medical literature supports the benefits of adjunctive stem cell therapy in bone regeneration and repair. Consistent with the findings of our review, which focused exclusively on craniofacial bone regeneration and demonstrated the effectiveness of stem cell therapy in managing mandibular fractures, previous studies have also reported significant benefits in the treatment of osteoporotic vertebral compression fractures and non-union of long bone fractures (Zhang et al., 2024; Thurairajah et al., 2021; Kaspiris et al., 2022; Yi et al., 2022).

Beyond their capacity for direct differentiation, stem cells also play a pivotal role in creating a pro-regenerative microenvironment. Through the secretion of a diverse array of bioactive factors — including cytokines, chemokines, and growth factors such as vascular endothelial growth factor (VEGF), FGF-2, and BMP-2 — stem cells modulate local inflammation, promote the formation of new blood vessels, recruit host progenitor cells, and orchestrate the intricate cellular interactions required for effective healing (Da Silva et al., 2025; Zheng et al., 2019). These paracrine effects are crucial for overcoming the hostile conditions often present in large or chronically inflamed defects, where impaired vascularity and persistent inflammation can otherwise compromise regeneration (Latimer et al., 2021; Valdivia et al., 2017). For instance, the immunomodulatory properties of MSCs enable them to suppress the production of pro-inflammatory cytokines such as tumor necrosis factor-alpha (TNF-α) and interlukin-1beta (IL-1β), thereby shifting the local environment from one that is destructive to one that is conducive to tissue repair (Valdivia et al., 2017; Zheng et al., 2019). Additionally, stem cell-derived factors can enhance matrix remodeling, restore the balance between matrix metalloproteinases (MMPs) and their inhibitors, and even exert antimicrobial effects that help protect regenerating tissues from infection (Bartold et al., 2019; Liu et al., 2019).

In addition to resulting in accelerated and enhanced abundance and volume of regenerated bone, the quality of regenerated bone is a crucial component of assessing success of regenerative therapies. As presented in many of the included studies (Pasquali et al., 2021; Pasquali et al., 2015; Aloise et al., 2018; Whitt et al., 2020), the regenerated bone following application of stem cells exhibits enhanced vascularity and compactness, both of which play critical roles in long-term success of any regenerative approach, specifically involving dental implants.

One of the main strengths of the present review is the inclusion of all prospective clinical studies with a minimum of five patients, regardless of surgical technique or treated anatomical site. This inclusive approach allowed for the presentation of a broad and integrative overview of stem cell use in diverse oral and craniofacial regenerative therapies. However, this comprehensiveness introduced substantial heterogeneity across the included studies in terms of design, patient population, types of stem cells utilized, therapeutic approaches, and follow-up durations. As a result, comparability across studies was limited, and pooled estimates require cautious interpretation. Subgroup analyses were conducted, where applicable, to address this variation. Additionally, this review exclusively evaluated regenerative outcomes of bone tissue within the craniofacial region. Extrapolation of these findings to other craniofacial tissues, such as cementum, or to non-craniofacial sites, such as long bones, requires further validation.

Of the 32 included RCTs and 27 NRSIs, only eight in each category were judged to have a low overall risk of bias. The main sources of bias were related to inappropriate random sequence generation and confounding. Although the number of studies with high risk of bias was relatively small—seven assessed with RoB2 and six with ROBINS-I—the reliance on studies with unclear risk of bias may have influenced the findings of this review. Despite including 59 studies, the considerable heterogeneity in outcome measures, study designs, and procedures prevented us from conducting a meta-regression to evaluate the effect of overall and domain-specific risk of bias on the effect size. Nevertheless, we performed sensitivity analyses by excluding studies with high overall risk of bias. Excluding studies with unclear or moderate risk was not feasible, as too few remained for meaningful analysis. With the exception of the meta-analysis on radiographic bone quality, all other analyses included studies with high overall risk of bias. While the results for CAL gain were not affected by excluding high-risk studies, the findings for both histologic bone quality and radiographic bone quantity were sensitive to their inclusion. When these high-risk studies were removed, only the effect of stem cell therapy on volumetric radiographic bone quantity remained statistically significant (Supplementary Figure S3). It is also important to note that we employed SMD using Hedges’ *g* as the summary statistic, which corrects for small sample bias and is inherently conservative. Applying an additional restriction by excluding studies with high risk of bias, on top of this conservative effect, may represent a double conservatism, making it particularly difficult to detect significant results. The pool of included studies makes it clear that future research on stem cell therapy in craniofacial bone regeneration should place greater emphasis on adhering to established standards for conducting clinical trials, particularly with respect to random sequence generation and allocation concealment.

To the best of the authors’ knowledge, this is the first systematic review and meta-analysis to evaluate the effects of stem cell therapy across the full spectrum of oral and craniofacial surgical procedures. Quantitative synthesis was performed to provide pooled effect estimates, offering a consolidated view of treatment outcomes. Due to the variability in measurement scales and reporting formats across studies, SMD using Hedges’ *g* was adopted instead of raw mean difference (MD) to enable meaningful comparisons across heterogeneous outcomes. The comprehensiveness of this review enables a broader understanding of how stem cell therapies are being applied clinically and the outcomes being reported. At the same time, it highlights ongoing challenges, particularly the need for greater consistency in study protocols, outcome measures, and reporting standards. Future investigations would benefit from standardized methodologies, including consistent definitions of clinical endpoints, rigorous stem cell characterization, and extended follow-up periods. Such efforts will enhance comparability, reduce heterogeneity, and improve the overall quality and applicability of evidence in this growing field of regenerative therapy.

## 5 Conclusion

Stem cell therapy has demonstrated promising outcomes in regenerative approaches targeting oral and craniofacial bones. However, heterogeneity in protocols and designs of the studies limits generalizability and impedes drawing robust conclusions. According to the results of our meta-analyses, stem cell therapy results in higher volume of regenerated bone, although in 2-dimensional planes—vertical and horizontal, it did not yield in better results. Also, meta-analysis showed that histologically stem cell therapy results in a regenerated bone with higher quality, however, this observation could not be confirmed in meta-analysis of radiographic evaluations. Finally, almost 75% of the studies included in this report had either moderate or high overall risk of bias, which further reduces the quality of evidence; therefore, conducting more high-quality clinical trials is necessary to determine the true potential and benefit of stem cell-based approaches for oral craniofacial bone regeneration.

## Data Availability

The original contributions presented in the study are included in the article/Supplementary Material, further inquiries can be directed to the corresponding author.

## Glossary

ALP: Alkaline phosphatase.
ASC: Adipose tissue stem cell.
ATA: Adipose tissue aspirate.
BFPMSC: Buccal fat pad mesenchymal stem cell.
BMA: Bone marrow aspirate.
BMAC: Bone marrow aspirate concentrate.
BMMSC: Bone marrow mesenchymal stem cell.
BMMNC: Bone marrow mononuclear cell.
BMNCC: Bone marrow nucleated cell concentrate.
β-TCP: Beta-tricalcium phosphate.
CAL: Clinical attachment level.
CBCT: Cone beam computed tomography.
CCT: Controlled clinical trial.
CGF: Concentrated growth factor.
CI: Confidence interval.
CT: Computed tomography.
DBBM: Deproteinized bovine bone mineral.
DEXA: Dual-energy X-ray absorptiometry.
DPSC: Dental pulp stem cell.
ePTFE: Expanded polytetrafluoroethylene.
FGF-2: Fibroblast growth factor 2.
GBR: Guided bone regeneration.
GTR: Guided tissue regeneration.
HAM: Human amniotic membrane.
HSC: Hematopoietic stem cell.
HU: Hounsfield unit.
IL-1β: Interleukin-1 beta.
LBG: Linear bone growth.
MD: Mean difference.
MFAT: Microfragmented adipose tissue.
MMP: Matrix metalloproteinase.
MMSC: Multipotent mesenchymal stem cell.
MNC: Mononuclear cell.
MRI: Magnetic resonance imaging.
MSC: Mesenchymal stem cell.
NA: Not applicable/available.
NRSI: Non-randomized studies of intervention.
PD: Probing depth.
PDL: Periodontal ligament.
PDLSC: Periodontal ligament stem cell.
PMSC: Periosteal mesenchymal stem cell.
PPD: Pocket probing depth.
PRF: Platelet-rich fibrin.
PRISMA: Preferred reporting items for systematic reviews and meta-analyses.
PROSPERO: International prospective register of systematic reviews.
PRP: Platelet-rich plasma.
RCT: Randomized controlled trial.
REML: Restricted maximum likelihood.
RoB2: Cochrane’s risk of bias tool version 2.
ROBINS-I: Risk of bias in non-randomized studies of interventions.
SBSC: Small blood stem cell.
SCG: Super cell glue.
SMD: Standardized mean difference.
SVF: Stromal vascular fraction.
TMD: Temporomandibular disorder.
TMJ: Temporomandibular joints.
TNF-α: Tumor necrosis factor-alpha.
TRC: Tissue repair cell.
VEGF: Vascular endothelial growth factor.
3D: Three-dimensional.
